# Non-invasive evaluation of energy loss in the pulmonary arteries using 4D phase contrast MR measurement: a proof of concept

**DOI:** 10.1186/1475-925X-12-93

**Published:** 2013-09-23

**Authors:** Namheon Lee, Michael D Taylor, Kan N Hor, Rupak K Banerjee

**Affiliations:** 1 Mechanical Engineering, School of Dynamics Systems, University of Cincinnati, 593 Rhodes Hall, ML 0072, Cincinnati, OH 45221, USA; 2 Cincinnati Children’s Hospital Medical Center, The Heart Institute, 3333 Burnet Avenue, Cincinnati, OH 45219, USA; 3 Pediatric Cardiology, The Heart Center, Nationwide Children’s Hospital, 700 Children’s Drive, Columbus, OH 43205, USA

**Keywords:** 4D phase contrast MRI, Pulmonary insufficiency, Energy-based endpoint

## Abstract

**Background:**

The repair surgery of congenital heart disease (CHD) associated with the right ventricular (RV)-pulmonary artery (PA) pathophysiology often left patients with critical post-operative lesions, leading to regurgitation and obstruction in the PAs. These lesions need longitudinal (with time) assessment for monitoring the RV function, in order for patients to have appropriate treatment before irreversible RV dysfunction occurs. In this research, we computed energy loss in the branch PAs using blood flow and pressure drop data obtained from 4D phase contrast (PC) MRI, to non-invasively quantify the RV-PA pathophysiology.

**Methods:**

4D PC MRI was acquired for a CHD patient with abnormal RV-PA physiology, including pulmonary regurgitation and PA stenosis, and a subject with normal RV-PA physiology. The blood velocity, flow rate, and pressure drop data, obtained from 4D PC MRI, were used to compute and compare the energy loss values between the patient and normal subjects.

**Results:**

The pressure drop in the branch PAs for the patient was −1.3 mmHg/s and −0.2 mmHg/s for the RPA and LPA, respectively, and was larger (one order of magnitude) than that for the control. Similarly, the total energy loss in the branch PAs for the patient, -96.9 mJ/s and −16.4 mJ/s, for the RPA and LPA, respectively, was larger than that for the control.

**Conclusions:**

The amount of energy loss in the pulmonary blood flow for the patient was considerably larger than the normal subject due to PA regurgitation and PA stenosis. Thus, we believe that the status of RV-PA pathophysiology for CHD patients can be evaluated non-invasively using energy loss endpoint.

## Background

With the excellent survival rate of palliated congenital heart disease (CHD) patients, monitoring residual lesions has become increasingly important [1,2]. Particularly, patients with right ventricular (RV) or pulmonary valve lesions, such as tetralogy of Fallot (TOF), aortic valve disease requiring the Ross procedure (aortic autograft with RV-PA homograft), or complex transposition of the great arteries (TGA), are often left with pulmonary insufficiency (PI) leading to progressive RV dilatation and occasionally resulting in pressure overload due to residual pulmonary stenosis. Those sequelae can result in progressive RV myocardial dysfunction, increasing the risk of sudden death [3-6].

Surgical or catheter-based pulmonary valve replacement is often required to rectify severe RV myocardial dysfunction [7]. Since the appropriate time for intervention is critical, the patho-physiology of the RV and the pulmonary arteries (PAs) has to be carefully monitored throughout the patient’s lifetime. However, due to the complexity of symptoms, it is sometimes difficult to accurately assess the disease progression with existing cardiac indices alone. Commonly used metrics include body-surface-area (BSA) indexed RV end-diastolic and end-systolic volumes (EDV_I_ and ESV_I_, respectively), ejection fraction, and RV end-systolic pressure (ESP).

Recently, energy-based endpoints have been investigated in our research group [8-10] to help determine timing for surgical interventions. In particular, BSA indexed RV stroke work (SW_I_) and energy transfer ratio (*e*_*MPA*_) between the RV and main PA (MPA) were proposed in our previous study to evaluate the hemodynamic status of the RV and the PA for repaired CHD patients. These energy-based endpoints had the advantage of incorporating RV volume, pressure data, and flow conditions in the PAs into one single index, and differentiated the hemodynamics of RV and PA of patients from those of normal subjects with statistical significance (*p* <0.05). In addition, energy-based endpoints correlated well with current indices, such as RV EDV_I_ and RV ESP [9]. Further, other energy-based endpoints, such as energy dissipation and power loss, also have been evaluated in single-ventricle (Fontan) physiology from other group [11-13].

However, energy-based endpoints require invasive pressure measurement, i.e., cardiac catheterization, which limits their applicability to only those patients undergoing catheterization. With recent development of 4D phase contrast magnetic resonance imaging (PC MRI), three dimensional and three directional velocity data over the cardiac cycle can be obtained for the conduits and chambers of the entire heart [14-16]. Therefore, the pressure data can be estimated non-invasively for any of the heart’s conduits and chambers by using the time varying 3D velocity vector field from 4D PC MRI data [16,17].

In this research we computed the pressure drop along the PA non-invasively, from the MPA to the branch PAs (RPA and LPA, right and left PA, respectively), using 4D PC MRI data. This enabled us to calculate energy loss in the branch PAs over the cardiac cycle leading to quantification of localized PI caused by obstruction in the RVOT and PA. The novelty of this research is that the use of 4D PC MRI will allow non-invasive assessment of energy loss in the RV-PA pathophysiology; thus, avoiding the need for catheterization. We believe that non-invasive assessment of pressure drop along the PAs and subsequent energy loss calculation in the PAs will help in longitudinal monitoring of RV-PA physiology for CHD patients. This allows accurate assessment of progression of the disease, which may lead to improving the timing of intervention, and patient outcomes.

## Methods

### Study population

Two subjects were considered for a comparison in this study as shown in Table 1: a normal volunteer (male, age: 29 years, weight: 70 kg, and heart rate: 79 beats/min) as a control and a patient (male, age: 19 years, weight: 129 kg, and heart rate: 81 beats/min) with aortic valve disease who underwent the Ross procedure. The control had normal RV-PA physiology, normal pulmonary valve function, and no stenosis was seen in the PAs. The patient had a conduit replacing RV-PA homograft and stenosis at the RPA; as a result, the patient had severe pulmonary regurgitation and a dilated MPA. This resulted in abnormal RV-PA physiology an uneven distribution of PA blood flow for the patient.

**Table 1:** Demographics of the subject in the study

	**Age**	**Sex**	**Weight**	**Heart rate**
	**(years)**		**(kg)**	**(bpm)**
Control (normal RV-PA physiology)	29	Male	70	79
Patient (abnormal RV-PA physiology)	19		129	81

### Data acquisition

**Figure 1:**
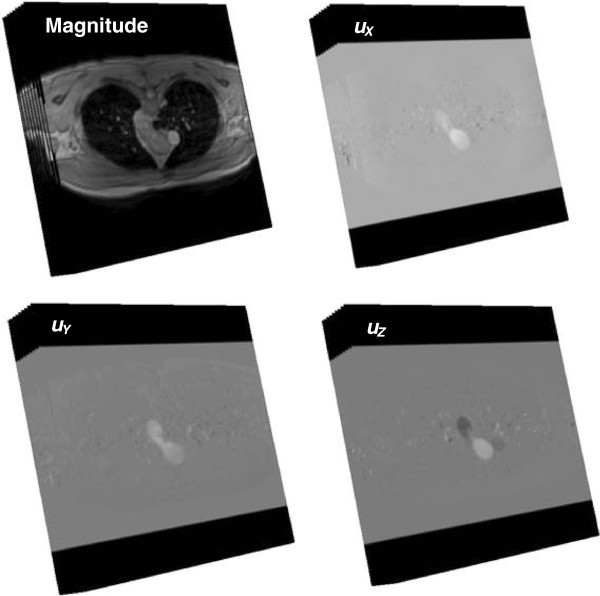
**The stack of PC MR images, magnitude images and three directional phase images- AP (anterior to posterior), RL (right to left), and FH (foot to head)- containing the velocity information, *u***_***x***_**(t),** ***u***_***y***_**(t),** ***u***_***z***_**(t) , respectively, for the PAs.** 24 and 40 phases during the cardiac cycle were recorded per each slice for the control and patient subjects, respectively.

4D PC MRI was performed for both the control and the patient subject using a 3.0 Tesla MRI scanner (Achieva, Philips Healthcare, Best, The Netherlands) in Cincinnati Children’s Hospital Medical Center (CCHMC). Three dimensional and three directional velocity encoded data were acquired over the cardiac cycle for both the subjects (Figure 1). As listed in Table 2, 24 phases per cardiac cycle were recorded with 24 and 40 slices per each phase for the control and the patient, respectively. The axial volume for 4D PC MRI for the control was 32.0 cm × 32.0 cm × 6.0 cm with the spatial resolution of 2.5 mm × 2.5 mm × 2.5 mm and that for the patient was 30.0 cm × 30.0 cm × 10.0 cm with the spatial resolution of 2.34 mm × 2.34 mm × 2.5 mm. The velocity encoding (VENC) for all three directions (*x, y, z*) was 200 cm/s and 580 cm/s for the control and patient, respectively. The repetition time was 3.79 ms and 4.27 ms, the echo time was 1.81 ms and 1.65 ms for the control and the patient, respectively. The flip angle was 5° for both the subjects.

**Table 2:** Detail of 4D PC MRI acquisition parameters

	**Control**	**Patient**
No. Phase	24	24
No. Slice	24	40
Spatial resolution (mm)	128 × 128	128 × 128
Pixel size (mm)	2.5× 2.5	2.34 × 2.34
Slice spacing (mm)	2.5	2.5
Acquisition volume (cm)	32.0 × 32.0 × 6.0	30.0 × 30.0 × 10.0
VENC (cm/s)	200	580
Repetition time (ms)	3.79	4.27
Echo time (ms)	1.81	1.65
Flip angle (°)	5	5

### Data analysis

**Figure 2:**
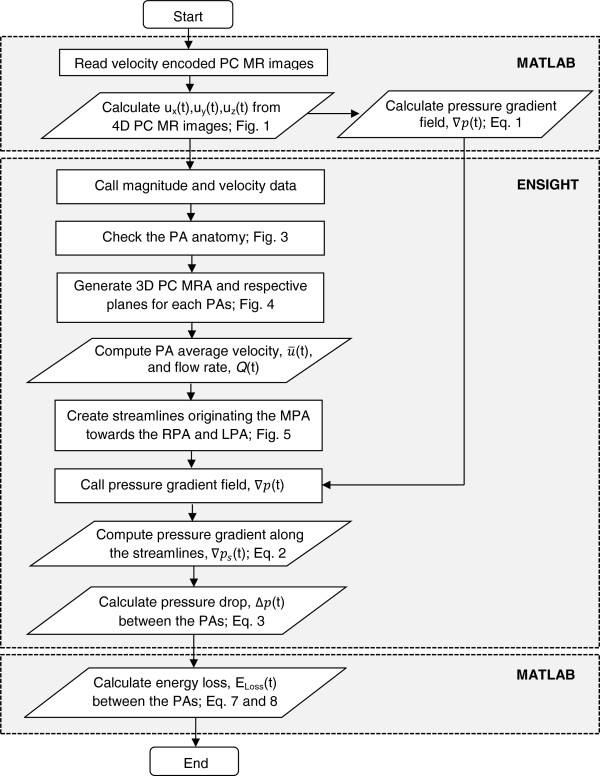
The flow chart to calculate the pressure drop and energy loss between the MPA and branch PAs using the velocity information obtained from 4D PC MR images.

The measured 4D velocity encoded PC MRI data was analyzed with an in-house MATLAB program (MATLAB, Inc., Waltham, MA) and semi-automated flow analysis software Ensight (CEI, Apex, NC), following the procedures presented in Figure 2. After reading 4D PC MR images, the transient blood velocity information, *u*_*x*_(t), *u*_*y*_(t), and *u*_*z*_(t), at each node was obtained from each directional phase images, AP (anterior to posterior), RL (right to left), and FH (foot to head), respectively. The pressure gradient field $\nabla P=\frac{\partial P}{\partial x_{i}}$, where *x* is the axial direction), which will be explained later, was calculated using the previously computed velocity information. Then, the magnitude of 4D MR image was converted to the geometry file, and the computed velocity and pressure gradient data (*∇P*) were converted to the variable files for further analysis in Ensight.

**Figure 3:**
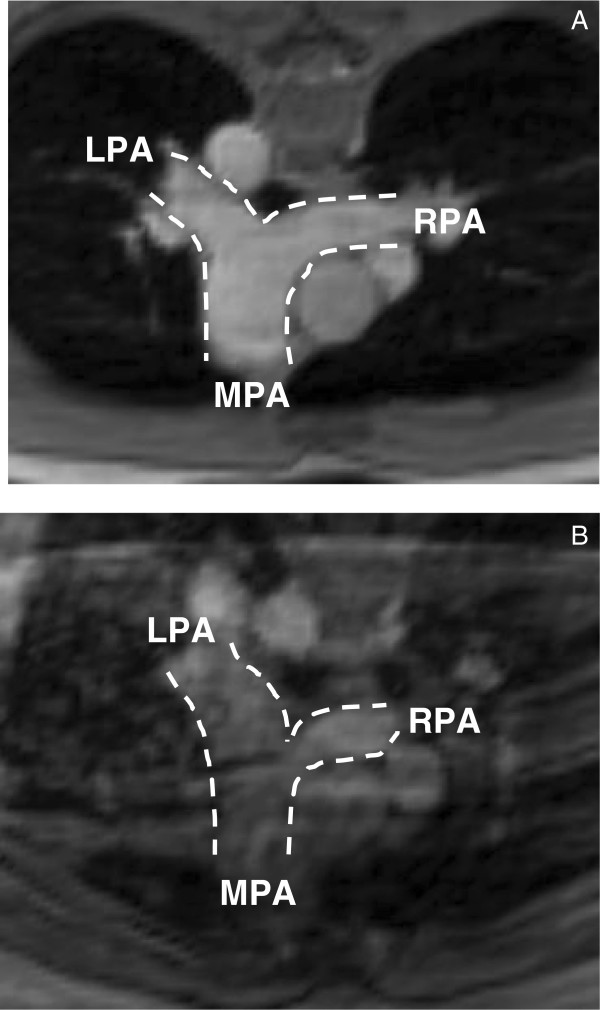
The detail of PA anatomy confirmed by the magnitude image of 4D PC MRI for the subjects, A) control and B) patient.

**Figure 4:**
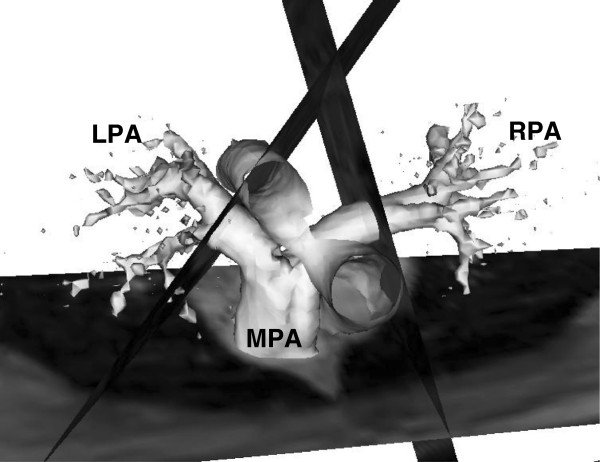
**3D image PA geometry for the control subject generated using the maximum velocity data at t = 0.096s with a semi-transparent iso-surface in Ensight.** The planes for the PAs, MPA, RPA, and LPA, are positioned perpendicular to the respective PAs.

**Figure 5:**
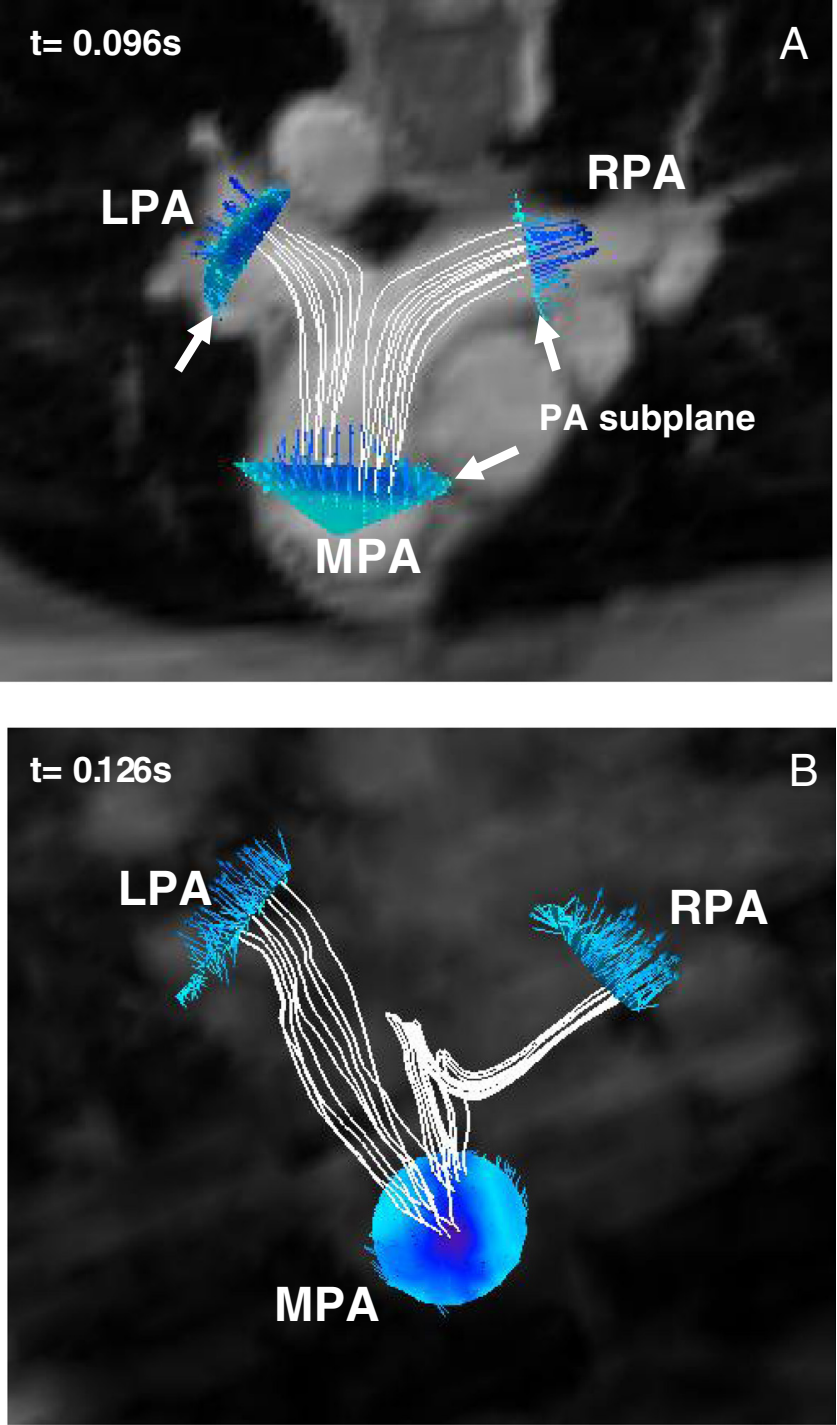
The subplanes of PAs for the flow computation and streamlines starting from the MPA for the pressure drop calculation are shown for the subjects, A) control and B) patient.

In Ensight, the PA anatomy for each subject was confirmed by magnitude images of the PA (Figures 3A and B for the control and the patient, respectively). Then, 3D image of PA geometry for each subject was constructed using 3D velocity data at the systolic phase and was visualized as a semi-transparent iso-surface. A representative 3D PA image of the control subject is shown in Figure 4. The MPA plane was placed approximately halfway between the pulmonary valve and the MPA bifurcation for the control subject. For the patient, the MPA plane was located at the distal end of the conduit, which assumed to be the origin of the MPA. Further, the planes for the branch PAs, RPA and LPA, were positioned at the location approximately 1 cm away from the first daughter branch of the RPA and LPA, to ensure that measured blood flow data were not affected by flow separation due to the bifurcation of PA’s daughter branches. The planes positioned perpendicular to the MPA, RPA, and LPA for creating subplanes, to measure the flow information in the PAs, are also shown in Figure 4. The subplanes were created on the respective planes covering the PA regions for computing the blood velocity and flow rate over the cardiac cycle at the PAs for the control (Figure 5A) and patient (Figure 5B).

**Table 3:** Diameter, average flow rate and velocity in the PAs for the control and patient

		**Diameter**	**Blood velocity**	**Blood flow**
		**(cm)**	**(cm/s)**	**(ml/s)**
Control	MPA	2.5	18.4	88.6
	RPA	1.5	25.9	47.7
	LPA	1.7	24.6	56.3
Patient	MPA	2.6	18.2	96.0
	RPA	2.0	15.6	48.5
	LPA	2.4	9.7	44.9

The streamlines of each pulmonary flow, MPA-RPA and MPA-LPA flows, and the velocity vectors in the respective subplanes during a systole phase are also shown in Figure 5. The streamlines that passed through the predominant portion of each pulmonary flow were chosen for the further pressure drop computation. The streamlines were started from uniformly distributed 3 × 3 seed points with a distance of approximately 2 mm on the plane. The time varying pressure drop calculation between the branch PAs and the MPA over the cardiac cycle was performed along those streamlines (described in the following section). Multiple streamlines were used for the pressure drop calculation since the calculated pressure drop may be dependent upon the selected streamline. Thus, the average pressure drop values for each pulmonary flow were reported in this study.

#### Pressure drop calculation

The time varying pressure drop between the branch PAs and the MPA (*dP* = P_Branch PA_ ‒ P_MPA_) can be calculated by integrating the pressure gradient along the streamline from the MPA to the branch PAs over the cardiac cycle [17]. As mentioned earlier, the pressure gradient field, $\nabla P\;\left(=\frac{\partial P}{\partial x_{i}}\right)$, was computed from the velocity information using the Navier–Stokes equation (Eq. 1). The flow is considered incompressible, unsteady, and laminar. Equation 1 was rearranged to solve the pressure gradient field, $\nabla P\;\left(=\frac{\partial P}{\partial x_{i}}\right)$ and *∇P* was calculated using a second-order central difference discretization method.

(1) $$\frac{\partial u_{i}}{\partial t}+u_{j}\frac{\partial u_{i}}{\partial x_{j}}=-\frac{1}{\rho }\frac{\partial P}{\partial x_{i}}+\frac{\mu }{\rho }\nabla^{2}u_{i}+F_{i}$$

where, *u* is blood velocity data, *ρ* the blood density (= 1,050 kg/m^3^), *μ* the blood viscosity (= 0.00345 Pa/s).

Since the streamline created was a 3D spline, each streamline was divided into 30 discrete points along the direction of streamline, generating 29 line segments connecting two adjacent points. The pressure gradient along the line segments on the streamline, $\nabla P_{s}\left(=\frac{\partial P}{\partial s_{n}}\right)$, was obtained by the dot product between the pressure gradient field $\left(\nabla P=\frac{\partial P}{\partial x_{i}}\right)$, calculated using Eq. 1b, and the normalized line vector of the line segment $\left({\vec{s}}_{{}_{n}}\right)$ as shown in Eq. 2.

(2) $$\nabla P_{s}\left(=\frac{\partial P}{\partial {\vec{s}}_{n}}\right)=\nabla P\cdot {\vec{s}}_{n}\;\text{where},n=1,2\ldots N$$

The pressure drop along the PAs (*dP*_Branch PA_), i.e., the pressure difference between two endpoints of the streamline, was calculated by integrating the pressure gradient along the streamline (Eq. 3), as mentioned earlier.

(3) $$dP_{\text{Branch}\;\mathrm{PA}}=P_{\text{Branch}\;\mathrm{PA}}-P_{\text{MPA}}=\int_{0}^{L}\frac{\partial P}{\partial s_{n}}\mathrm{ds}\;\text{where},L=\text{length}\;\mathrm{of}\;a\;\text{streamline}$$

Thus, there are two *dP*_Branch PAs_; one is *dP*_RPA_ and the other is *dP*_LPA_.

The calculation procedures (Figure 2) to compute pressure drop were verified with the pressure drop computed for a simplified 2D stenosis model using a finite difference CFD solver (Fluent, ANSYS, Inc., Canonsburg, PA, USA). The detail of the verification procedures and results are provided in the Appendix section.

#### Energy loss calculation

The rate of total energy loss in the branch PAs $\left({\dot{E}}_{\text{Loss},\text{Branch}\;\text{PAs}}\right)$ in this study was defined as the difference in the rate of total energy transferred between the branch PAs and the MPA, resulting in two terms, *major* and *minor* energy losses, as shown in Eq. 4.

(4) $${\dot{E}}_{\text{Loss},\text{branch}\;\text{PAs}}={\dot{E}}_{\text{Loss},\text{major}}+{\dot{E}}_{\text{Loss},\text{minor}}$$

The ${\dot{E}}_{\text{Loss},\text{major}}$ is the difference in the rate of the pressure-flow and kinetic energies transferred between the branch PAs and the MPA as shown in Eq. 5a:

(5a) $${\dot{E}}_{\text{Loss},\text{major}}=\left({\dot{E}}_{\text{RPA}}+{\dot{E}}_{\text{LPA}}\right)-{\dot{E}}_{\text{MPA}}$$

(5b) $${\dot{E}}_{\text{RPA}}={Ρ}_{\text{RPA}}\cdot Q_{\text{RPA}}+\frac{1}{2}\rho {\bar{u}}_{\text{RPA}\;}^{2}Q_{\text{RPA}}$$

(5c) $${\dot{E}}_{\text{LPA}}=P_{\text{LPA}}\cdot Q_{\text{LPA}}+\frac{1}{2}\rho {\bar{u}}_{\text{LPA}}^{2}\;Q_{\text{LPA}}$$

(5d) $${\dot{E}}_{\text{MPA}}=P_{\text{MPA}}\cdot Q_{\text{MPA}}+\frac{1}{2}\rho {\bar{u}}_{\text{MPA}}^{2}\;Q_{\text{MPA}}$$

The ${\dot{E}}_{\text{Loss},\text{major}}$ (Eq. 5a) can be rewritten in terms of pressure drop, flow rate, and velocity at the PAs. The right-hand side of Eq. 5b, 5c, and 5d, i.e., the rate of the pressure-flow and kinetic energy terms at the PAs [9], RPA, LPA and MPA, respectively, can be substituted into Eq. 5a while maintaining flow balance at the PAs (*Q*_*MPA*_ = *Q*_*RPA*_ + *Q*_*LPA*_). As a result, the revised the equation for ${\dot{E}}_{\text{Loss},\text{major}}$ becomes Eq. 5e:

(5e) $$\begin{array}{l} {\dot{E}}_{\text{Loss},\text{major}}=dP_{\text{RPA}}\cdot Q_{\text{RPA}}+\frac{1}{2}\rho \left({\bar{u}}_{\text{RPA}}^{2}-{\bar{u}}_{\text{MPA}}^{2}\right)\cdot Q_{\text{RPA}}+dP_{\text{LPA}}\cdot Q_{\text{LPA}}\\ \;+\frac{1}{2}\rho \left({\bar{u}}_{\text{LPA}}^{2}-{\bar{u}}_{\text{MPA}}^{2}\right)\cdot Q_{\text{LPA}} \end{array}$$

where, *dP*_*BranchPA*_ is the time varying pressure drop in the branch PA computed previously. The first two terms of the right-hand side of Eq. 5e account for the rate of pressure-flow and kinetic energy losses in the RPA, respectively, and the next two terms represent those energy losses in the LPA.

The ${\dot{E}}_{\text{Loss},\text{minor}}$ in Eq. 4 is the rate of the energy loss in the branch PA due to flow separation at the MPA bifurcation. The minor energy loss at each branch PA, ${\dot{E}}_{\text{Loss},\text{minor}}$, can be computed using a flow resistance coefficient (ζ) derived based on the ratio of the flow rate at the PA (=*Q*_*Branch PA*_/*Q*_*MPA*_) [18] as shown in Eq. 6.

(6) $${\dot{E}}_{\text{Loss},\text{minor}}=\zeta_{\text{Branch}\;\text{PA}}\frac{1}{2}\rho {\bar{u}}_{\text{Branch}\;\text{PA}}^{2}\;Q_{\text{Branch}\;\text{PA}}$$

Therefore, the final form of the equation we used to calculate the rate of total energy loss in each branch PA $\left({\dot{E}}_{\text{Loss},\text{Branch}\;\text{PA}}\right)$ becomes Eq. 7:

(7) $$\begin{array}{l} {\dot{E}}_{\text{Loss},\text{Branch}\;\text{PA}}=dP_{\text{Branch}\;\text{PA}}\cdot Q_{\text{Branch}\;\text{PA}}+\frac{1}{2}\rho \left({\bar{u}}_{\text{Branch}\;\text{PA}}^{2}-{\bar{u}}_{\text{MPA}}^{2}\right)\cdot Q_{\text{Branch}\;\text{PA}}\\ \;+\zeta_{\text{Branch}\;\text{PA}}\cdot \frac{1}{2}\rho {\bar{u}}_{\text{Branch}\;\text{PA}}^{2}\cdot Q_{\text{Branch}\;\text{PA}} \end{array}$$

The net total energy loss in the branch PA $\left({\dot{E}}_{\text{net}\cdot \text{Loss},\text{Branch}\;\text{PA}}\right)$ over the cardiac cycle (*T*) was computed by integrating the rate of total energy loss in the PA (${\dot{E}}_{\text{Loss},\text{Branch}\;\text{PA}}$; Eq. 7) over *T* as shown in Eq. 8.

(8) $$E_{\text{net}\;\text{loss},\text{Branch}\;\text{PA}}=\int_{0}^{T}{\dot{E}}_{\text{Loss},\text{Branch}\;\text{PA}}\left(t\right)\text{dt}\;$$

In this study, the patient had abnormal RV-PA physiology compared to the control subject. To highlight this, the PA diameters measured from 4D MRI along with the spatially averaged blood velocity and flow rate at each PAs are presented in the following results section. Subsequently, the results for non-invasively computed pressure drop and energy loss in the branch PAs for the control and the patient are provided.

## Results

**Figure 6:**
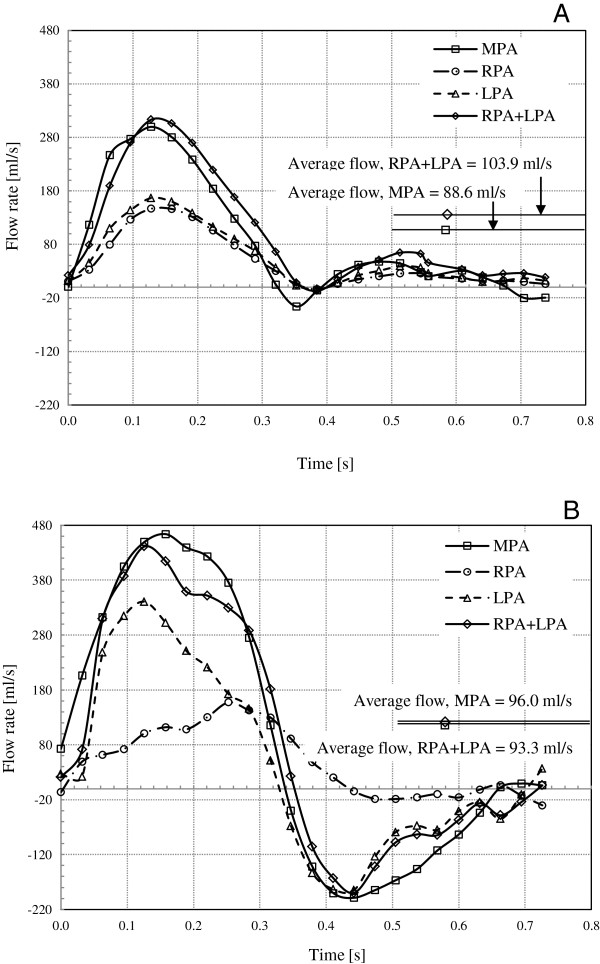
**The blood flow rate versus time curves for the subjects. A)** The average flow volumes for the control are 88.6 ml/s and 103.9 ml/s, for the MPA and the RPA + LPA, respectively. **B)** The average flow volumes for the patient are 96.0 ml/s and 93.4 ml/s, for the MPA and the RPA + LPA, respectively.

**Figure 7:**
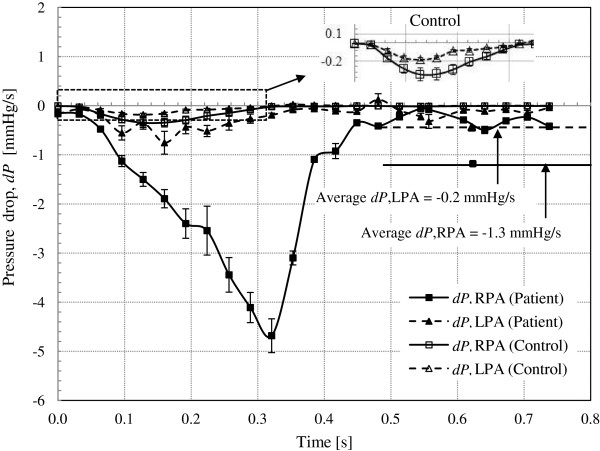
**The pressure drop along the branch PAs versus time curves for the subjects.** The average pressure drop in the branch PAs for the patient was larger than the control, -1.3 mmHg/s and −0.2 mmHg/s, for the RPA and LPA, respectively. Whereas, the pressure drop along the branch PAs for the control was minimal. The enlarged image of a part of the pressure drop curves for the control is presented.

**Figure 8:**
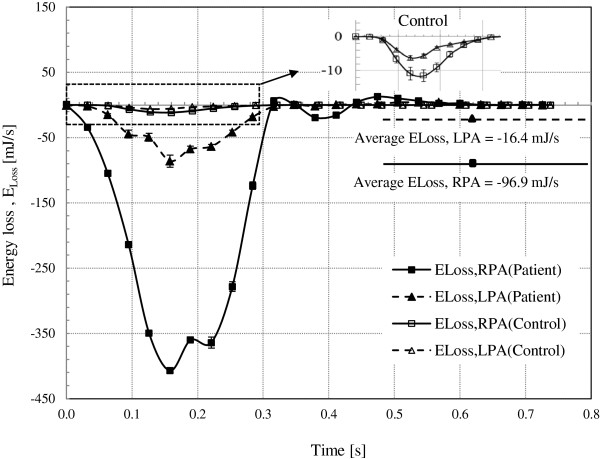
**The energy loss along the branch PAs versus time curves for the subjects.** The average energy loss in the branch PAs for the patient was larger than the control, -96.9 mJ/s and −16.4 mJ/s, for the RPA and LPA, respectively. The enlarged image of the energy loss curves for the control is given.

Table 3 shows the computed time averaged blood velocity and flow rate at the PAs for both the patient and control subject. The flow rates versus time curves for each PA are presented in Figure 6. The computed values of pressure drop and energy loss in the branch PAs are presented in Table 4. The pressure drop and energy loss in the branch PAs versus time curves for both subjects are shown in Figures 7 and 8, respectively.

**Table 4:** Average and peak pressure drop, and average and net total energy loss in the PAs for the control and patient

	**Control**		**Patient**	
	**RPA**	**LPA**	**RPA**	**LPA**
Ave. pressure drop (mmHg/s)	−0.09	−0.04	−1.3	−0.2
Peak pressure drop (mmHg)	−0.4	−0.2	−4.7	−0.8
Ave. total energy loss (mJ/s)	−2.1	−1.1	−96.9	−16.4
Net total energy loss (mJ)	−1.6	−0.8	−70.3	−11.9

### Time averaged blood velocity and flow rate at the PAs

The time averaged blood velocities at the PAs for both the subjects are presented in Table 4. The time average blood velocities for the control were 18.4 cm/s, 25.9 cm/s, and 24.6 cm/s for the MPA, RPA, and LPA, respectively. The time average blood velocity at the MPA was lower than that at the branch PAs, because the area of MPA is larger (diameter of the MPA, D_MPA_ = 2.5 cm; Table 4) compared to the sum of the areas of the two branch PAs (D_RPA_ = 1.5 cm and D_LPA_ = 1.7 cm). The time average blood velocities for the patient were 18.2 cm/s, 15.6 cm/s, and 9.7 cm/s for the MPA, RPA, and LPA, respectively. The time average blood velocities at the PAs for the patient were lower than the control due to the negative flow in the patient’s PAs. Further, the average blood velocity was lower at the LPA than the RPA due to considerably larger regurgitation that occurred at the LPA during the diastole phase of the pulse for the patient.

In Figure 6, the blood flow rate versus time curves at the PAs for both the subjects are shown. The control had the average blood flow volumes of 88.6 ml/s, 47.7 ml/s, and 56.3 ml/s for the MPA, RPA, and LPA, respectively (Figure 6A and Table 4). The summation of RPA and LPA average flow was 103.9 ml/s and was somewhat more than the MPA average blood flow (88.6 ml/s) by 17.3%. The small amount of reverse blood flow in the MPA was observed during the early and late diastole phases. No reverse flow was observed in the branch PAs, RPA and LPA.

In contrast, the time average blood flow volumes for the patient were 96.0 ml/s, 48.5 ml/s, and 44.9 ml/s for the MPA, RPA, and LPA, respectively (Figure 6B and Table 4). The summation of RPA and LPA flow was 93.4 ml/s and was within 2.7% of the MPA flow. As shown in Figure 6B, the large amount of reverse blood flow in the MPA and the LPA was observed during diastole phase. During the early systole over 70% of blood from the MPA directly flows to the LPA; however, the blood flow in the RPA increased with time until it reached the late systole phase. Reverse flow in the RPA was insignificant during the diastole phase.

### Pressure drop along the branch PAs

Figure 7 shows the pressure drop between the branch PA and the MPA (*dP*_*Branch PA*_ = P_Branch PA_ − P_MPA_) versus time. The average pressure drop values at any instant of time calculated from nine streamlines for each pulmonary flow was used to construct the time varying pressure drop curves with standard error. As presented in Table 3, the average pressure drop was minimal in the control (−0.09 mmHg/s and −0.04 mmHg/s, for the RPA and LPA, respectively) compared to that of the patient (−1.3 mmHg/s and −0.2 mmHg/s, for the RPA and LPA, respectively). Similarly, the peak pressure drop was smaller for the control (−0.4 mmHg and −0.2 mmHg, for the RPA and LPA) than that for the patient (−4.7 mmHg and −0.8 mmHg, for the RPA and LPA).

### Energy loss in the branch PAs

The rate of total energy loss along the branch PA (${\dot{E}}_{\text{Loss},\text{Branch}\;\text{PA}}$) versus time curves are shown in Figure 8. The average total energy loss was larger for the patient (−96.9 mJ/s and −16.4 mJ/s, for the RPA and LPA, respectively) compared to that for the control (−2.1 mJ/s and −1.1 mJ/s, for the RPA and LPA). Also, the net total energy loss in the PAs over the cardiac cycle was much larger for the patient (−70.3 mJ and −11.9 mJ, for the RPA and LPA) than that for the control (−1.6 mJ and −0.8 mJ, for the RPA and LPA).

## Discussion

Pulmonary regurgitation, residual RVOT and PA obstruction are the main post-operative lesions seen in many cases of repaired CHD [2,6,19]. The abnormal RV-PA hemodynamics, resulting from pulmonary regurgitation and residual obstruction, directly affect the efficiency of pulmonary blood flow. Further, chronic pulmonary regurgitation and PA obstruction can cause progressive RV volume and pressure overload resulting in RV dilatation and hypertrophy. As these symptoms worsen, RV dysfunction can occur, leading to often sudden death.

**Figure 9:**
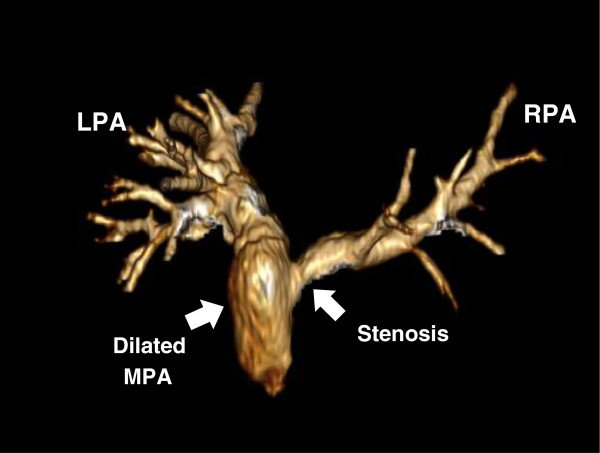
3D representative of gadolinium-enhanced magnetic resonance angiography (Gd-MRA) of PA for the patient showing stenosis at the RPA origin and the dilated MPA that caused an uneven PA flow distribution.

The patient in this study underwent the Ross procedure resulting in the replacement of the pulmonary valve with a conduit. Lack of a competent valve causes pulmonary regurgitant flow (pulmonary regurgitation fraction, a ratio between the backward and forward blood volumes, = 36.5%) and adverse PA physiology. In addition, the stenosis at the RPA origin (Figure 9) causes large pressure drop in the RPA leading to a highly uneven PA flow distribution. As confirmed in Figure 6B, the MPA-LPA flow is much higher than the MPA-RPA flow during the early systole phase. Most of the blood volume from the MPA rapidly flows into the LPA during early systole, whereas the MPA-RPA flow increases with time until the late systole. Thus, the flow in the MPA-RPA is out of phase with respect to the MPA-LPA flow. Both severe pulmonary regurgitation and the blood overflowing in the MPA cause its dilation in case of the patient (Figure 9). Consequently, the MPA-RPA flow of the patient becomes irregular compared to that of the control (Figure 6B). A lesser volume of blood directly flows to the RPA; whereas, most of the blood flowing to the RPA became trapped, swirled, and recirculated in the dilated MPA (see Figure 5B) for a short period of time (30 ~ 40 ms), then flowed into the RPA along a tortuous path. However, the PA flows of the control were evenly distributed as shown in Figures 5A and 6A.

The patient had significantly larger energy loss compared to the control subject as shown in Figure 8. The average energy loss in the patient was −96.9 mJ/s and −16.4 mJ/s, for the RPA and LPA, respectively. These values for the patient are one order of magnitude higher than those for the control subject (Table 3). The energy loss in the RPA of the patient was particularly higher due to the stenosis as mentioned in the previous paragraph. The higher pressure drop in the patient leads to the increased energy loss in the branch PAs, as shown in Figure 8.

The additional energy loss due to blood flow separation in the MPA bifurcation, ${\dot{E}}_{\text{Loss},\;\text{additional}}$ in Eq. 7, which is the minor energy loss from the perspective of a fluid mechanics, was calculated by the multiplication of a flow resistance coefficient (ζ) and kinetic energy loss and was added to the total energy loss. A flow resistance coefficient (ζ) was determined based on the ratio of flow rates in the PAs, *Q*_*Branch PA*_/*Q*_*MPA*_, for each time point during the cardiac cycle [18]. The contribution by minor energy loss to the total energy loss was less significant for the control subject (2.1% and 4.1% for the RPA and LPA, respectively) than the patient (19.0% and 7.1% for the RPA and LPA, respectively). Particularly, the minor energy loss for the patient’s RPA was relatively large (19.0%) due to flow obstruction in the RPA caused by the stenosis.

Inherent noise exists on phase contrast velocity mapping due to the motion of PAs during the contraction and relaxation of the RV [20]. It is observed that noise resulting from PA movement was manifested near the boundary of artery. Therefore, the streamlines which passed adjacent to the PA boundary were not considered in computing the pressure drop along the PA.

The elastic energy storage in the MPA was estimated to be about 1% of the total energy transferred to the MPA [10]. The majority of the energy transfer occurs through the form of the pressure-flow energy and the kinetic energy, as discussed in our previous study [10]. Thus, elastic energy loss in the branch PAs would be insignificant. However, other possible energy losses in the branch PAs, such as friction loss between blood and tissues as blood flows in the PAs and local effects due to narrowing or stenosis in the daughter branches of the branch PAs, are difficult to measure under a clinical setting. Therefore, the energy loss calculation in this study may somewhat underestimate the actual energy loss in the branch PAs while such losses need to be assessed in future. We believe such losses are order of magnitude lower than pressure-flow and kinetic energy losses, which are accounted for in this study.

The accurate measurement of cardiac pressure data is necessary for the previously proposed energy-based endpoints, such as RV stroke work index (SW_I_) and energy transfer ratio (*e*_*MPA*_). However, diagnostic cardiac catheterization is not a part of standard care for typical CHD patients. Therefore, continuous quantification and monitoring of the change in the pressure using catheterization for a patient is not trivial. In this regard, 4D PC MRI has an advantage as it can provide 4D (*u, v, w, t*) velocity information needed to compute pressure drop, flow, and energy-based endpoints non-invasively [16]. Further, the methodology used for this research does not include a complicated numerical computation, such as computational fluid dynamics (CFD), which is time consuming and requires significant lead time to pre-process, compute, post process, and analyze the numerical data. Therefore, the proposed technique would extend the clinical applicability of the energy-based endpoints for longitudinal assessment of patient on a regular basis. A study that extends the proposed methodology and involves statistically relevant control subjects and CHD patients is needed. We believe such a study would reveal the significant difference in the pressure drop and energy loss in the branch PAs between control subjects and CHD patients.

## Conclusions

We have used non-invasive 4D PC MRI data to calculate the energy loss in the branch PAs and compared between a CHD patient with abnormal PA physiology and a normal subject with normal PA physiology. Based on our results, the patient had considerably larger energy loss in the branch PAs due to pulmonary regurgitation and PA obstruction compared to the normal subject. Therefore, we believe that non-invasively obtained energy loss in the branch PAs can be a useful clinical measure for evaluating the longitudinal changes of RV-PA pathophysiology of repaired CHD patients.

### Nomenclature

CHD = congenital heart disease, RV = right ventricle, PA = pulmonary artery, 4D PC MRI = 4 dimensional phase contrast magnetic resonance imaging, TOF = tetralogy of Fallot, TGA = transposition of the great arteries, PI; pulmonary insufficiency, BSA = body surface area, EDV = end-diastolic volume [ml], ESV = end-systolic volume [ml], SW = stroke work [J], MPA = main pulmonary artery, RPA = right pulmonary artery, LPA = left pulmonary artery, *e*_*MPA*_ = energy transfer ratio, CFD = computational fluid dynamics, CCHMC = Cincinnati Children’s Hospital Medical Center, VENC = velocity encoding, AP = anterior to posterior, RL = right to left, FH = foot to head, *∇P* = pressure gradient field, MRA = magnetic resonance angiogram, *u* = blood velocity [cm/s], *Q* = blood flow rate [ml/s], *ρ* = blood density [kg/m^3^], *μ* = blood viscosity [Pa/s], *dP* = pressure drop [mmHg], ${\dot{E}}_{\text{Loss},\text{branch}\;\text{PA}}$, = the rate of energy loss in the branch PA [mJ/s], ${\dot{E}}_{\text{Loss},\text{major}}$ = the rate of major energy loss [mJ/s], ${\dot{E}}_{\text{Loss},\text{minor}}$ = the rate of minor energy loss [mJ/s], $\;{\dot{E}}_{\text{PA}}$ = the rate of energy in the PA [mJ/s].

## Appendix

### The verification of the pressure drop computation

Physiologic pressure data in the subject's PA was not available because catheterization was not performed in this study. Thus, the pressure drop calculation in this study was verified with CFD result using a simplified geometry as following.

### Computation model

**Figure 10:**
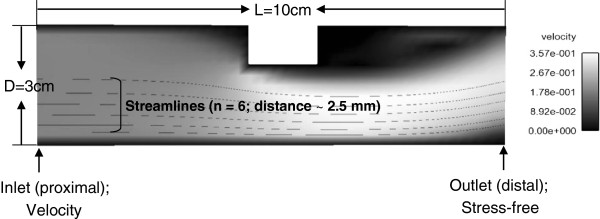
**The computation model used in CFD analysis for the verification of pressure drop computation.** The difference in the pressure drop between two methodologies was 7.3%.

A 2D geometry (length of 10 cm and depth of 3 cm; Figure 10) with a blockage in the middle section was created in GAMBIT. After mesh generation with the resolution of 2.5 mm × 2.5 mm, which is the same as the one that 4D PC MR images have in the study, the mesh was exported from GAMBIT for CFD analysis. The CFD analysis was performed in finite volume solver (FLUENT, ANSYS, Inc., Canonsburg, PA, USA) assuming the blood to be Newtonian fluid with the viscosity of 3.45 cp (= 0.00345 Pa/s) and the density of 1,050 kg/m^3^. The average MPA blood velocity, (0.137 m/s) for the control subject in the study was applied at the inlet. At the outlet boundary, a stress-free boundary condition was used.

### Pressure drop computation

After CFD analysis, the 2D velocity information was extracted from the converged solution. The pressure gradient field ($\nabla P=\frac{\partial P}{\partial x_{i}}$) was computed using the extracted velocity field (Eq. 1). Then, in Ensight six streamlines originating from the inlet were generated with a distance of approximately 2.5 mm and the pressure gradient along each streamlines ($\nabla P_{s}=\frac{\partial P}{\partial s_{n}}$) was obtained (Eq. 2). The pressure drop at the outlet was computed by integrating the pressure gradient along each streamline (Eq. 3). The average pressure drop value was obtained from six streamlines (0.22 mmHg) using the proposed method (Figure 2) and was compared with the pressure drop computed from CFD solution (0.24 mmHg). The difference in the pressure drop between two methodologies was 7.3% [= (0.24-0.22)/0.24 × 100].

## The comparison of pressure drop and energy loss in the branch PAs

As mentioned the previous section, the validation of our results with physiologic pressure drop and energy loss in the LPA and RPA was not possible since catheterization was not performed for the subjects in this study. Alternatively, the pressure drop and energy loss in the branch PA were calculated for two subjects from our previous study (10), who underwent catheterization and 2D cardiac MRI in their PAs separately, and were compared to our results. The CHD patient had severe pulmonary regurgitation and moderate stenosis in the LPA, and normal subject had normal RV-PA physiology. Both are comparable to the subjects in this study. As shown in Table 5, the average pressure drop and energy loss in the branch PA from this study were in the similar range with the physiologic pressure drop and energy loss values calculated from clinical data. Although the patient in this study had lower pressure drop in the RPA (−1.3 mmHg/s) than the patient’s LPA in the previous study (−3.7 mmHg/s), the total energy loss in the RPA (−96.9 mJ/s) was higher than that in the LPA (−41.9 mJ/s) because the kinetic energy loss was considerably large due to imbalanced blood flows in the patient's PA of this study.

**Table 5:** Comparison of average pressure drop and total energy loss in the branch PA in this study with physiological data

			**Pressure drop** **[mmHg/s]**	**Total energy loss** **[mJ/s]**
Normal	RPA	4D PC MRI	−0.09	−2.1
		Physiological data	−0.12	−3.7
Patient	RPA	4D PC MRI	−1.3	−96.9
	LPA	Physiological data	−3.7	−41.9

## Competing interests

The authors declare that they have no competing interests.

## Authors’ contributions

NL carried out study design, data analysis and interpretation, and drafting of the manuscript. RB’s contributions include study design, data interpretation, and approval of the final manuscript. KN performed MR acquisition. MT carried out MR acquisition and participated in study design, data interpretation. All authors read and approved the final manuscript.
